# Application of CO_2_-Laser Micro-Perforation Technology to Freeze-Drying Whole Strawberry (*Fragaria ananassa Duch*.): Effect on Primary Drying Time and Fruit Quality

**DOI:** 10.3390/foods13101465

**Published:** 2024-05-09

**Authors:** Marlene Pinto, Cynthia Kusch, Karyn Belmonte, Silvana Valdivia, Pedro Valencia, Cristian Ramírez, Sergio Almonacid

**Affiliations:** 1 Departamento de Ingeniería Química y Ambiental, Universidad Técnica Federico Santa María, Valparaíso 2390123, Chile; marlene.pinto@usm.cl (M.P.); cynthia.kusch@alumnos.usm.cl (C.K.); karyn.belmonte.12@sansano.usm.cl (K.B.); silvana.valdivia.12@sansano.usm.cl (S.V.); cristian.ramirez@usm.cl (C.R.); 2 Centro de Biotecnología Daniel Alkalay Lowitt, Universidad Técnica Federico Santa María, Valparaíso 2390136, Chile; pedro.valencia@usm.cl

**Keywords:** freeze-drying, strawberry, CO_2_ laser micro-perforations, processing time and quality

## Abstract

Freeze-drying (FD) processing preserves foods by combining the most effective traditional technologies. FD conserves the structure, shape, freshness, nutritional/bioactive value, color, and aroma at levels similar to or better than those of refrigerated and frozen foods while delivering the shelf-stable convenience of canned/hot-air-dehydrated foods. The mass transfer rate is the essential factor that can slow down the FD process, resulting in an excessive primary drying time and high energy consumption. The objective of this study was to reduce the FD processing time using CO_2_ laser technology to improve product competitiveness in the preservation of whole strawberries. The research process consisted of the selection and characterization of fresh strawberries, followed by preparation, pre-treatment, freeze-drying, a primary drying time assessment, and a quality comparison. Experiments were carried out using strawberries without micro-perforation and with five and eight micro-perforations. Quality parameters were determined for fresh, frozen/thawed, and freeze-dried/rehydrated strawberries. It was found that the primary drying time can be significantly reduced by 20% (95% CI) from 26.7 h for non-perforated fruits to 22.3 h when five micro-perforations are made on each strawberry. The quality parameters used to evaluate the strawberries did not show significant differences when comparing frozen/thawed fruits with freeze-dried/rehydrated fruits. The experiments conducted in this study showed that freeze-drying may efficiently compete with freezing technology when processing whole strawberries.

## 1. Introduction

Freeze-drying (FD) processing preserves foods by combining the most effective traditional technologies. FD conserves the structure, shape, freshness, nutritional/bioactive value, color, and aroma at levels similar to or better than those of refrigerated and frozen foods while delivering the shelf-stable convenience of canned or hot-air-dehydrated foods [1,2,3]. Moreover, the FD process removes most of the water from food via sublimation; thus, a significant weight reduction is achieved, which provides ease of handling and lower shipping/storage costs together with a long-term shelf life. Nonetheless, despite its unsurpassed advantages, FD has always been recognized as the most expensive process for manufacturing food products. The high cost is due to both the required capital investment in vacuum technology and the running expenses. In a typical FD process, a completely frozen food product is placed into a freeze-drying chamber, where the sublimation or primary drying stage starts. The mass transfer rate is the essential factor affecting the speed of the FD process. A low mass transfer rate results in an excessive primary drying time and concomitant high energy consumption, increasing the process cost. It has been estimated that the basic energy requirement for removing 1 kg of water from food under FD technology is at least double that for the conventional air-drying method, and the cost of FD is 4–8 times higher [4,5,6,7,8,9]. Today, the food industry faces an increasingly competitive market that requires producers to improve productivity by optimizing the invested capital, energy utilization, and processing time. For example, expensive equipment, such as that used in FD systems, should be used intensively by reducing the batch time [10]. While FD research dates back some 20 years, the advances necessary to enable broader commercial adoption are still lacking, particularly regarding developments to significantly reduce the processing time and associated energy expenditure. These factors underpin lower-cost production, which would unlock significant market potential and facilitate access to better pro-health products for a larger spectrum of people of all income levels [11]. In this direction, a microwave field was applied as a heat source in FD, which proved, at a laboratory scale, that the drying efficiency and energy requirement can be enhanced. However, as reviewed by the authors in [12], there are still some problems in the application of the microwave field method at the technological/industrial scale. From a mass transfer point of view, the main effect on sublimation time is a result of the product resistance, which is determined by the porous dried zone and depends on the porosity and thickness of the food. There have been numerous approaches to managing mass transfer resistance. The first approach is to control the pore size by means of the freezing stage. This method establishes the shape and size of the ice crystals, which later affect the mass transfer rates during the subsequent sublimation and desorption stages [7,13,14]. Another approach is the controlling of the Initial Nucleation Temperature (Ti_N_), which could be used to obtain a frozen product with a specific structure, later resulting in a uniform drying behavior. The induction of nucleation through ultrasonic technology stimulates nucleation near the system melting point, generating large and practically vertical ice crystals, leading to a very high permeability in the sublimation stage [15]. The authors of [16] reported that the drying time was reduced from 30 to 16 h when the previous freezing was carried out using the vacuum-induced surface method instead of spontaneous freezing. Nonetheless, the quality—mainly the shape, texture, and rehydration—of the final product was significantly affected; moreover, these laboratory-scale technologies have failed to be scaled up because of their technological complexities [15,16]. It should be noted that, in these cases, it was reported that large and needle-shaped ice crystals produced pores that significantly reduced the drying time, suggesting that a type of crystal with this particular shape/size would effectively enhance the sublimation rate during the primary drying stage.

An alternative option is the application of micro-perforation by means of CO_2_ laser technology, which is an established technology in various fields owing to its superior process accuracy, environmental cleanliness, and safety. CO_2_ laser technology permits perforations to be made at precise locations on multiple surfaces and arrangements within a size range of 50 to 300 μm in diameter [17], and it may be able to reproduce the needle-shaped spacing that has been reported to have positive results [15]. Some reports detail experimental applications of CO_2_ laser beams to foodstuffs to accelerate mass transfer processes; for example, the authors of [18] used this technology to create channels in wheat seeds to enhance water penetration in order to accelerate tempering [18]. The authors of [19] perforated blueberry skin using CO_2_ laser technology to improve mass transfer during osmotic dehydration–infusion. Recently, in our laboratory, this technology was used to drill tomato skin to accelerate lay infusion in the process of tomato peeling [20]. This superficial modification resulted in an increase in the diffusivity values by 2.3-fold compared with those of unmodified surfaces [20]. More recently, also in our laboratory, CO_2_ laser micro-perforations were applied to modify the skin resistance during blueberry FD with the aim of avoiding the characteristic blueberry-bursting phenomena; the percentage of non-bursting units was improved from 47 to 86%, and the primary drying time was reduced by 25% [21]. However, the effect of this method on the primary drying time of freeze-dried strawberries has not been studied, even though strawberries constitute one of the most widely grown berries globally and are consumed fresh, frozen, or freeze-dried [22]. 

CO_2_ laser technology also represents an important component of the food industry’s overall environmental strategy because its energy efficiency could be improved, especially when considering the complete cycle of processing, transportation, and storage. It was estimated that the ratio of FD to freezing costs for peas is reduced from 1.36 to 1.19 if frozen storage is considered when computing the costs [23]. Therefore, if the primary drying time can be reduced, the advantages of FD technology over freezing technology could be increased, especially for whole strawberries. The general objective of the present study was to reduce the FD processing time by using CO_2_ laser technology to improve product competitiveness in the preservation of whole strawberries, especially compared with whole frozen strawberries. The specific objectives of this study were to determine the effect of CO_2_ laser micro-perforations on the primary drying time and to determine and compare the quality parameters of fresh strawberries and strawberries subjected to different treatments: freezing/thawing and freeze-drying/rehydrating.

## 2. Materials and Methods

**Figure 1:**
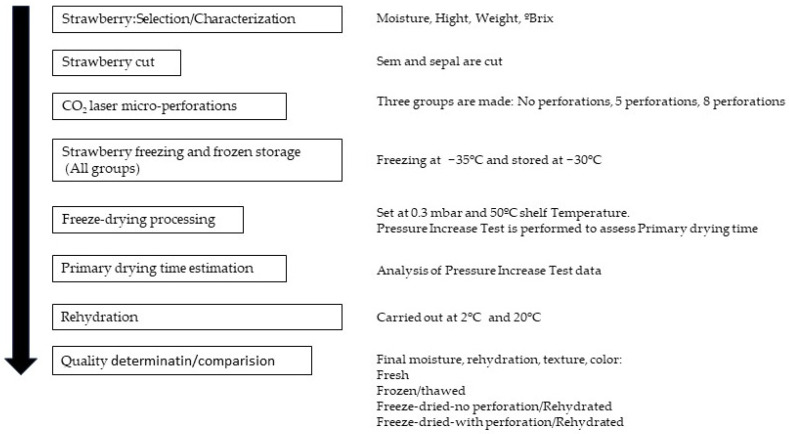
General procedure flow.

The research process employed in this study consisted of the selection and characterization of fresh strawberries, followed by preparation, pre-treatment (perforation), freeze drying, primary drying time assessments, quality determinations, and comparisons. Figure 1 shows the general procedure, which is then explained in detail.

### 2.1. Selection and Preparation of Strawberry Fruits

**Figure 2:**
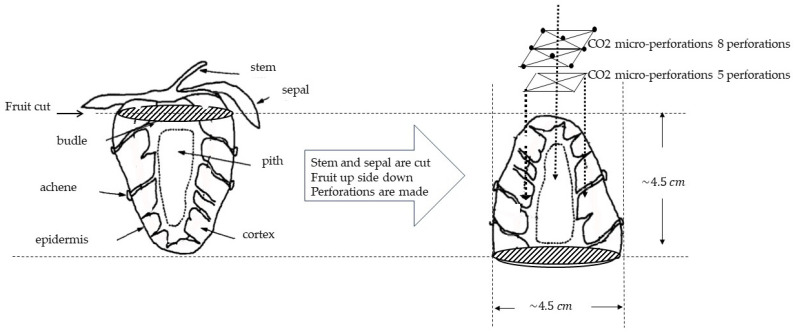
Strawberry diagram showing cut area and CO_2_ laser micro-perforation patterns.

The experiments were carried out using fresh strawberries of the variety *Fragaria ananassa Duch*, which were purchased from a local market. Strawberries with an approximate length of 4.5 cm were selected, and the stems/sepals were cut, which resulted in a circular base area with a diameter of approximately 4.5 cm (Figure 2).

To ensure the homogeneity of the raw material used for the experimental treatments, the strawberries were characterized by their Total Soluble Solids (TSS) content, also known as the °Brix value [24], which was selected as one of the main quality indicators for the strawberry fruit. To determine the °Brix value, the strawberry fruits were weighed using a RADWAG balance (AS 220/C/2, Radom, Poland). Next, they were crushed and homogenized with 10 mL of distilled water for 5 min until a homogeneous juice was obtained. The resulting juice was filtered, and aliquots were taken for °Brix analysis using an HI 96,680 digital refractometer (Hanna Instruments, Smithfield, RI, USA). Finally, a dilution scale-down calculation was made to obtain the °Brix value of the basic undiluted juice.

### 2.2. CO_2_ Laser Micro-Perforations and Laser System Settings

In general, the process of laser perforation of a product to be pre-treated can be carefully regulated by adjusting the parameters of speed and laser power; in this way, it is possible to configure the laser with extreme accuracy according to the desired results. The perforation treatment of the strawberries was carried out using a 100 W CO_2_ laser system (Firestar t100, Synrad Inc., Mukilteo, WA, USA). The system was implemented with a 125 mm focusing lens (FH series Flyer, Synrad Inc.) and a computer interface with laser marking software (WinMark Pro, V6 Synrad Inc.). A distance of 128 mm was fixed between the laser and the surface of each strawberry. The system was run at a continuous wavelength of 10.6 μm and 10 kHz frequency. Fruits with five, eight, and no perforations were prepared. When forming 5 perforations, a square grid pattern of 2.0 × 2.0 cm centered on the tip of the fruit cone shape was made. For 8 perforations, the same grid was used by adding 3 perforations and adjusting the center to the tip of the cone (see Figure 2). The CO_2_ laser was set at 120 pulses, a duration of 1 millisecond, a linear speed of 100 cm/s, and a percentage of power that was determined experimentally by observing (Helmut Hund GmbH, H600/12, Wetzlar, Germany) the depth of the perforation using a microscope until a laser configuration that allowed us to perforate at least one-third of the fruit height was achieved by varying the power of the laser (maximum 100 watts).

In order to observe the structure of the micro-perforated strawberry, a microstructural analysis was carried out using a Carl Zeiss Evo MA 10 scanning electron microscope (SEM) (Carl Zeiss SMT, Ltd., Cambridge, UK) equipped with an energy-dispersive X-ray spectroscopy detector (X-ACT, Oxford Instruments, Oxford, UK) [25]. The dimensions of the perforations and the degree of tissue damage caused by the laser burn were observed. The tissue porosity was especially noted to ensure that the walls of the perforations were not calcinated so that water vapor could still flow from the strawberry through the perforations. After the perforation treatment, the samples with and without perforations were frozen at −35 °C to a homogenous temperature of −30 °C and then maintained at that temperature until the freeze-drying stage.

### 2.3. Determination of Primary Drying Time

After freezing, the FD process proceeds to the primary drying or sublimation stage. The primary drying stage—conventionally the most time-consuming part of the process—was investigated in the present study. All experiments were carried out in a Martin Christ freeze-dryer, model Alpha 2-4 LSCplus (Martin Christ Gefriertrocknungsanlagen, Osterode, Germany), which can operate at a total vacuum pressure of up to 0.001 mbar, monitored/controlled with a MKS Baratron 622 capacitance manometer (MKS Instruments, Andover, MA, USA) and a condenser that can operate at temperatures down to −85 °C. It has three 0.021 m^2^ shelves that can each be temperature-controlled with a wireless temperature monitoring system, which, in turn, allows for sample temperature monitoring through pt100 port sensors. The strawberry samples were placed onto the three shelves, considering a total load of 9 units (3 per shelf). In order to assess the product temperature, a miniature pt100 sensor (PT 100 Mini-Epsilon LSC Plus, Martin Chris, Osterode am Harz, Germany) was inserted into one unit. A standard freeze-drying processing condition was fixed at 0.3 mbar (30 Pa) with a condenser temperature of −85 °C and a shelf temperature of 50 °C. This shelf temperature was chosen by considering quality optimization studies on freeze-dried strawberries from the related literature [2,26,27]. The freeze-dryer system includes dedicated software (SCADA Software V1 LPC Plus Martin Chris, Osterode am Harz, Germany) to set, control, and monitor the freeze-drying process under the selected processing conditions. In addition to the shelf temperature and vacuum pressure, the Pressure Increase Test (PIT), a widespread technique used for monitoring the primary drying time, was performed: the valve placed in the spool connecting the vacuum chamber to the condenser was closed every 30 min for 30 s. While this valve was closed (30 s), the pressure inside the chamber increased because of the accumulation of water vapor. The chamber pressure data were collected during the PIT, and the percentage increase was automatically computed. At the beginning, the pressure was at a high value, and then it reached a maximum before decreasing as the sublimating vapor ran out. The primary drying time was considered to be completed when the percent rise was equal to or less than 10%. SCADA Software LPC plus software allowed for the graphic visualization and recording of several process variables: the tray temperature, strawberry temperature, condenser temperature, chamber absolute pressure (capacitive sensor), and PIT data.

### 2.4. Determination of Quality

#### 2.4.1. Moisture Content

The moisture content of the product was determined by using AOAC method no. 934.06. An amount of 5 to 10 g of the sample was dried under vacuum conditions in an oven at 70 °C for 24 h. The moisture of the sample was determined using Equation (1) [28]:

(1) $$Moisture\;content\;\left(\%\right)=\frac{Mass\;initial-Mass\;of\;the\;dry}{Masss\;initial}*100$$

The moisture content of the product was determined at the end of the primary drying and freeze-drying (subsequent secondary drying) stages.

#### 2.4.2. Rehydration

Strawberries with and without micro-perforations were compared by analyzing two rehydration temperatures (2 and 20 °C). First, the freeze-dried strawberries were brought to room temperature. Rehydration of the strawberries was carried out according to the process described in [29], with modifications. Each strawberry was immersed in 100 mL of distilled water for 1, 2, 3, 4, 5, 10, 15, 20, 25, 30, 35, 40, 45, 50, 55, or 60 min. After this, the samples were allowed to drain to eliminate excess water on the outside of the strawberries, and the fruits were weighed. Finally, the rehydration value was obtained according to Equation (2), where *W_r_* is the weight after rehydration and *W*_0_ is the initial weight of the strawberry. The analyses were carried out in triplicate.

(2) $$Rehydration\;\left(\%\right)=\frac{W_{r}}{W_{0}}*100$$

#### 2.4.3. Color

The color of the samples was determined according to the method described in [30] using a Konica Minolta colorimeter (model CR-410, Tokyo, Japan). With this device, the coordinates of the uniform color space CIEL*a*b* were measured. The type of illuminant was D65, and the observer degree was 2° [28]. The measurements were carried out in triplicate, obtaining the average of five measurements in the equatorial plane per strawberry. 

The tone angle parameter hue and chroma [27] were obtained according to Equations (3) and (4), respectively.

(3) $${hue}_{ab}=Arctg\left(\frac{a^{*}}{b^{*}}\right)$$

(4) $${Chroma}_{ab}=\sqrt{{\left(a^{*}\right)}^{2}+{\left(b^{*}\right)}^{2}}$$

In the color analysis, fresh, thawed, and freeze-dried/rehydrated strawberries with and without micro-perforations were compared.

#### 2.4.4. Mechanical Test

The texture test was carried out according to the method described in [31], with modifications, using a Brookfield (Brookfield, CT, USA) texturometer (or texture analyzer). A comprehension test was carried out on the strawberries using the TA11/1000 probe with an activation load of 0.07 N, a test speed of 1 mm/s, and a deformation of 30%. This test measures the force necessary to produce a given deformation and/or the deformation caused by a given force to obtain the force required to compress a food between the molars or the tongue and palate. The texture measurement test was carried out on rehydrated and thawed strawberries in triplicate.

### 2.5. Statistical Analysis

All data are reported as the means of three replicates and their respective standard deviations. Significance tests were performed via analysis of variance (ANOVA) and Duncan’s multiple range tests with a significance of 95% using STATGRAPHIC Centurion XVIII software, Statpoint Inc.^®^ (Warrenton, VA, USA), 2018.

For the analysis of the Pressure Increase Test (PIT) vs. FD time data, the best fit of an exponential curve was regressed, and 95% CI bands around the fitted curve were included using STATGRAPHIC Centurion XVIII software, Statpoint Inc.^®^, 2018. 

## 3. Results and Discussion

### 3.1. Strawberry Characteristics

Strawberries with a height of approximately 4.5 cm were selected, obtaining an average of 4.51 ± 0.39 cm over 50 units. After the stem and sepal of each strawberry were cut, a circular base of approximately 4.5 cm was obtained. The average mass (*n* = 50) was calculated to be 30.0 ± 4.4 g. The Total Soluble Solids contents of the strawberries used as raw material for the experimental procedures were within a narrow range of 7.1 ± 0.5 °Brix, which was in agreement with the °Brix value of 7° recommended by the UC Davis Postharvest Research and Extension Center [32]. The moisture content was determined to be 89.1 ± 2%.

### 3.2. CO_2_ Laser Micro-Perforation

Micro-perforation was performed as described in the methodology: a 2.0 cm × 2.0 cm square grid of perforations was applied, and at least one-third of the strawberry depth was penetrated by utilizing 50% power (50 W). The final settings of the laser and the resulting perforations are shown in Table 1. It can be observed that the visual impact of the perforation treatment was negligible as the strawberry surface had a high density of “achene” components or seeds (see Figure 1) that camouflaged the laser perforation effect. Moreover, the perforations penetrated deep into the strawberry pith, which is the cavity that contains an important amount of water (or ice, when frozen). Finally, a pronounced strawberry aroma was noted, so any odor as a result of perforations was not detected.

### 3.3. Freeze-Drying Processing

Freeze-drying processing was carried out as described in the methodology. The processing conditions were a shelf temperature, chamber pressure, and condenser temperature of 50 °C, 0.3 mbar, and −85 °C, respectively. Three groups of strawberries were processed: those without perforations and those with five or eight micro-perforations. All tests were performed in triplicate.

**Figure 3:**
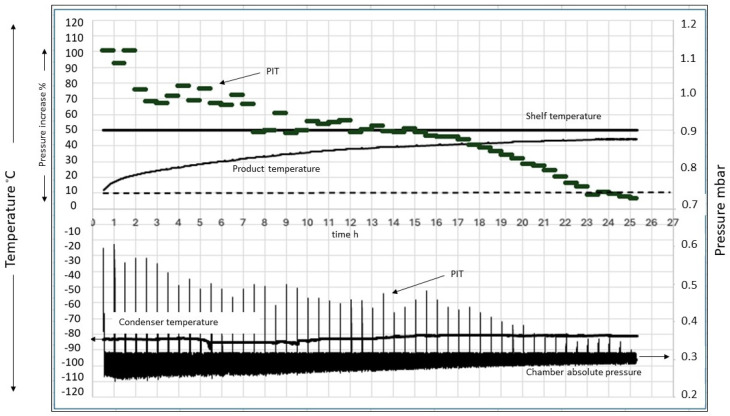
Example of freeze-drying process information.

Dedicated control software was used to monitor all set variables, and it was programmed to perform the Pressure Increase Test (PIT) every 30 min. Figure 3 depicts an example of the information registered during the freeze-drying process.

### 3.4. Primary Drying Time Assessment

**Figure 4:**
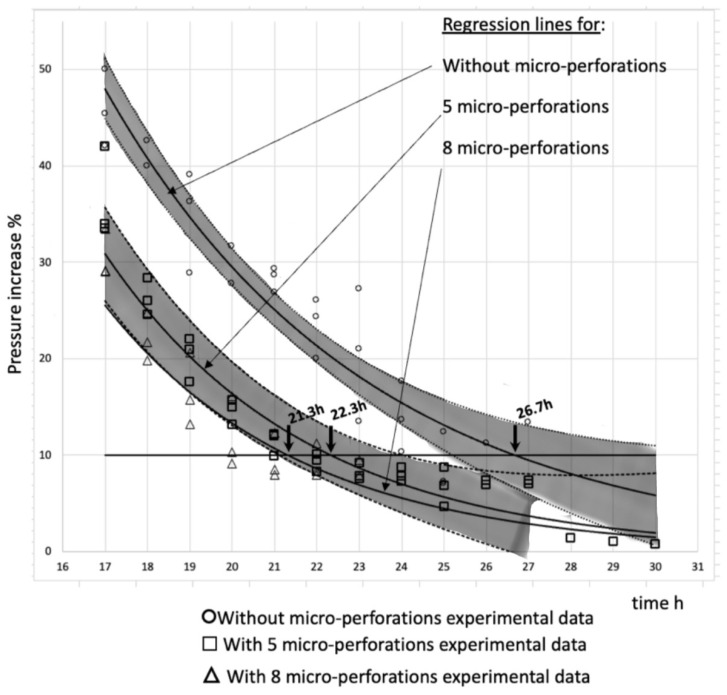
Treated data used to determine the primary drying time. Values were obtained at the intersections of the regression, lower, and upper lines of 95% CI bands with the 10% pressure increase line.

After all three groups of strawberries were freeze-dried (three times each group), the PIT data were extracted for further processing. As expected, the percentage pressure increase (PIT) value was high at the beginning of the process until around 2.0 h, in agreement with that shown in Figure 3. Then, the value descended for 1 h and remained approximately constant for 4 h. The value subsequently decreased at a moderate rate until 17 h, when it began to descend at a higher rate and, finally, reached a very low value, continuing to decline to values under 10% after around 23 h. This behavior was approximately similar in all cases, and at around 17 h in all experimental runs, the PIT value descended at a high rate until it crossed the threshold value of 10%. Taking this observation into account, the PIT data after 17 h of processing were used to assess the end of the primary drying time for the processing of strawberries without perforations and for those with five or eight perforations. An exponential line was regressed for data after 17 h to better assess the intersection with the 10% PIT threshold. Figure 4 shows the treated data including the experimental measurements of the PIT value with the respective regression lines (exponential), along with the upper and lower lines of the 95% confidence intervals, for strawberries without micro-perforations and for those with five micro-perforations. CI lines for the regression for strawberries with eight micro-perforations are not shown to avoid line overlap.

The primary drying time is indicated by the mean and corresponding lower and upper limits of the 95% CI. The values were obtained from the data in Figure 4.

Table 2 presents the mean, lower, and upper values of primary drying times, taken from the data set, the regression lines, and the respective upper and lower 95% CI lines. These FD times, between 20.5 and 27.1 h, are comparable to the times obtained in [27]: 30 h for whole strawberries freeze-dried at 27 °C or 24 h for those at 50 °C. However, much more information would be required (i.e., on the size of the fruits, the heat and mass transfer surface, and the total volume of fruits relative to the void volume of the FD chamber, among others) to conduct an exact comparison.

From Figure 4 and Table 2, it can be concluded that there was a significant difference between the primary drying time without micro-perforations (average 26.7 h) and the corresponding time when five micro-perforations were made in the strawberries (average 22.3 h); this is a difference of 4.4 h, or a 20% time reduction. In a basic analysis, if one month of operations takes 24 × 30 h = 720 h, five more batches could be processed with the same equipment. This is an important result if high-value products are being produced and higher productivity (through the more intensive use of high-cost equipment) is required.

The data also showed no statistical difference between treatments with five and eight micro-perforations. Based on this result, the quality parameters were analyzed for only the no-perforation and five-micro-perforation strawberries.

**Figure 5:**
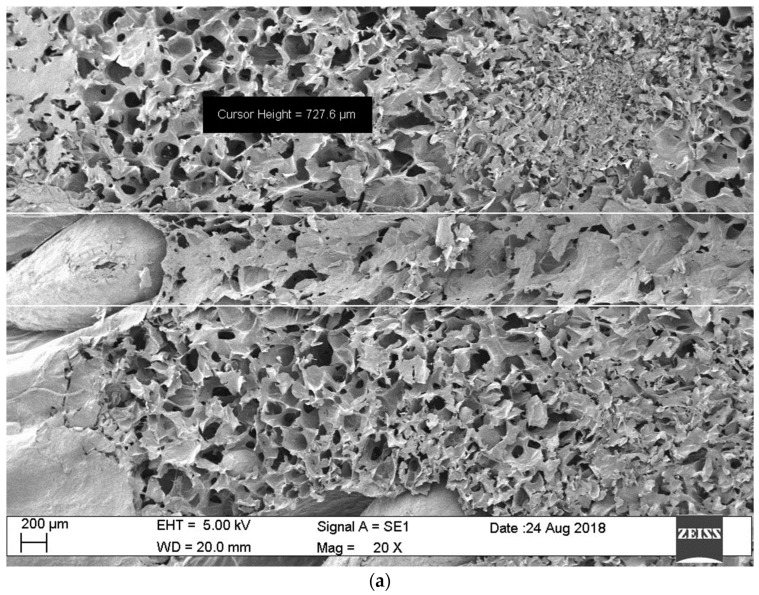

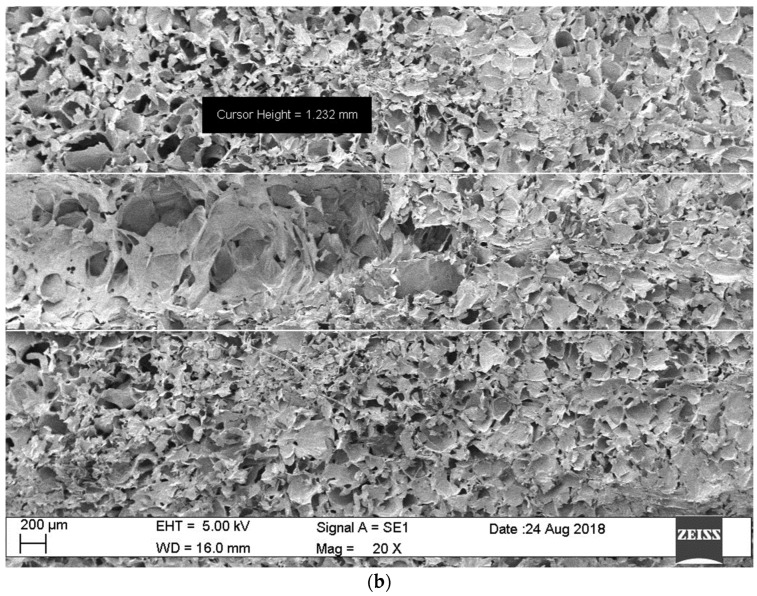
(**a**) Scanning electron microscopy (SEM) overview of the pore cavity within the strawberry tissue: initial diameter visualization. (**b**) Scanning electron microscopy (SEM) overview of the pore cavity within the strawberry tissue: final diameter visualization.

To explain the time reduction as an effect of micro-perforation, scanning electron microscopy (SEM) images of the micro-perforations were obtained. These images provide information on the perforation dimensions and the tissue matrix characteristics. The time reduction could be a result of a mass transfer enhancement produced by increasing the surface area and/or reducing the mass transfer resistance within the dried fraction of the strawberry tissue, facilitating the flow of water vapor out of the fruit. The SEM images are depicted in Figure 5a,b.

**Figure 6:**
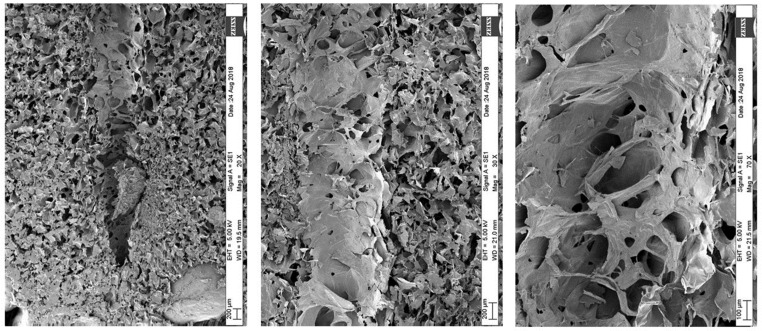
Scanning electron microscopy (SEM) overview of the pore cavity within the strawberry tissue: visualization of pore wall perforation.

The micro-perforations had a truncated cone shape with initial and final diameters of 0.060 cm and 0.125 cm. Figure 5a,b show the difference in diameter for the same perforation. With this information and the dimensions of the strawberry fruit (Figure 2), it can be calculated that the increase in strawberry surface area due to the perforations is approximately 1%, which is not significant. The other possible explanation is a mass resistance reduction, which is highly possible if the walls of the perforations are sufficiently permeable and not significantly affected by calcination from the laser beam. The integrity of the tissues is depicted in SEM images in Figure 6. It can be observed that pore cavities remained open, allowing for vapor flow. In addition, it should be noted that the distance to be traveled by the sublimated water became shorter as a result of the perforations, further reducing the mass transfer resistance.

### 3.5. Quality Analysis

#### 3.5.1. Rehydration 

The rehydration of the freeze-dried strawberries was used as a quality parameter related to the microstructure of the food matrix. The percentage of rehydration was calculated to indicate the degree of rehydration with respect to the fresh product. Rehydration was carried out at 2 and 20 °C. The color and texture (hardness) were also analyzed and compared for fresh, thawed, and rehydrated strawberries without and with five micro-perforations. The rehydration results are summarized in Table 3.

Low rehydration percentages were achieved, with not even 40% of the water lost through freeze-drying being restored. This factor is important since the process is expected to result in a food like the initial one (fresh), which was not obtained with this rehydration method. However, despite the low values, the strawberries with micro-perforations had better rehydration; this is because the micro-perforations increased the contact area of the strawberries, facilitating mass transfer. It should be noted that the CO_2_ laser did not close the pores through which the light beam passed, leaving the interior of the strawberry in direct contact with the water during the rehydration process (see Figure 4). Another influencing factor is the reduction in the primary drying time, since treating the strawberry for a shorter time at a high temperature produced less collapse in the structure, thus maintaining a porous product that helped water enter the food.

#### 3.5.2. Color

**Figure 7:**
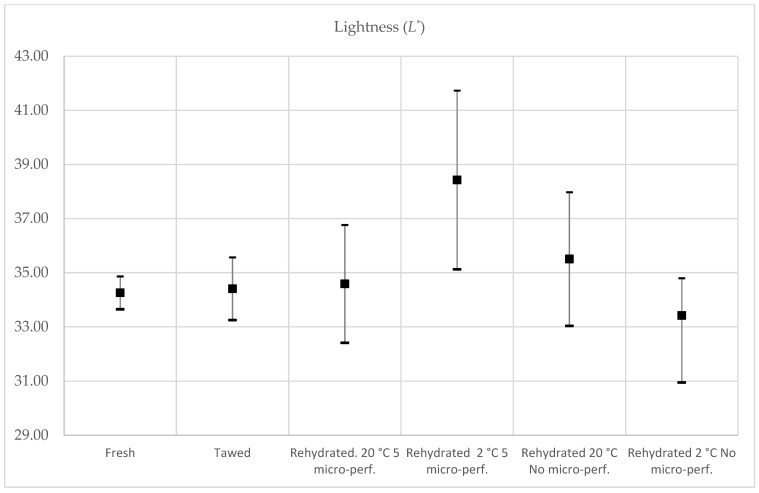
Lightness test results, 95% CI. Average value, maximum and minimum value are represented.

The color of the rehydrated freeze-dried fruit was compared with that of fresh and thawed strawberries. The luminosity (L*), green-to-red hue (a*), blue-to-yellow hue (b*), hue angle (hue), and purity of color (chroma) were determined. The results are shown in Table 4; the same letters in the same column indicate no statistical difference (95% confidence). The parameters of the fresh strawberries agree with those found in the literature for all the analyzed parameters [1]. In the case of the L* parameter, the rehydrated strawberries at 2 °C with micro-perforations obtained the highest value; additionally, their values were not within the range of fresh strawberries but were within the range of reliability of thawed strawberries, which means that they were not statistically different. In general, the L* parameter (Figure 7) for all rehydrated strawberries managed to fall within the confidence interval for the thawed strawberries, meaning that there was no significant difference between thawed strawberries and rehydrated strawberries.

**Figure 8:**
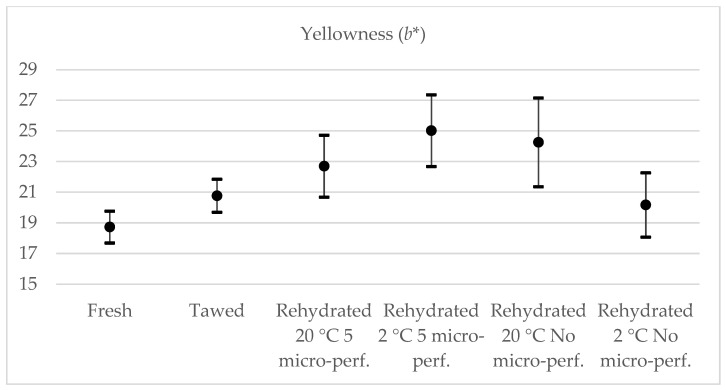
Yellowness test results, 95% CI. Average value, maximum and minimum value are represented.

**Figure 9:**
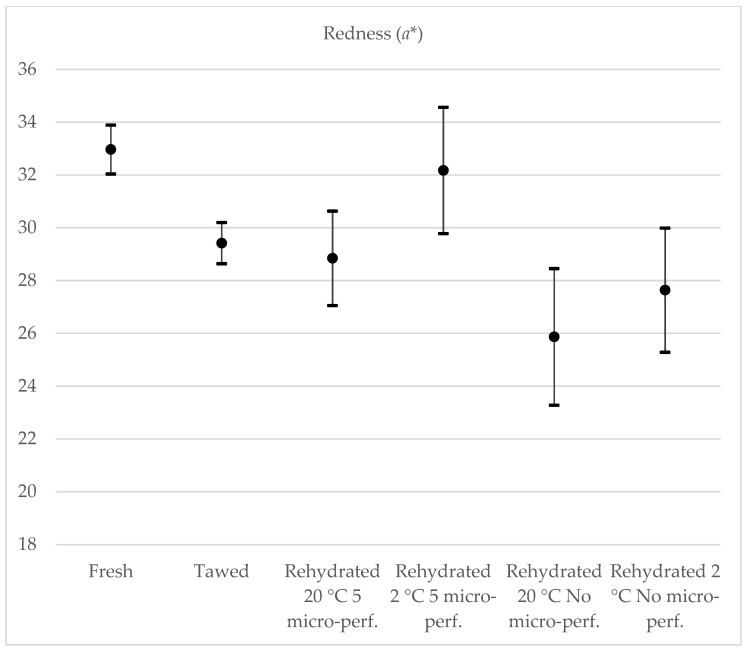
Redness test results, 95% CI. Average value, maximum and minimum value are represented.

When analyzing the shade from green to red (a*) (Figure 8), only strawberries rehydrated at 2 °C with five micro-perforations were part of the confidence interval for fresh strawberries; therefore, significant changes occurred in the tone of the a* color during freeze-drying and rehydration. However, there were no differences between rehydrated strawberries and thawed strawberries. When comparing the shade from blue to yellow (b*) (Figure 9), only those strawberries without micro-perforations rehydrated at 2 °C were the same as fresh strawberries; the others were not part of their confidence interval. However, only in strawberries rehydrated at 2 °C were significant differences found.

#### 3.5.3. Texture

**Figure 10:**
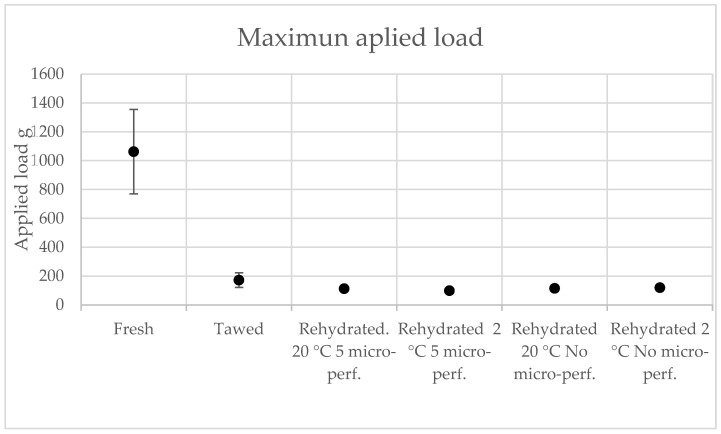
Texture test results, 95% CI. Average with standard deviation.

The texture of the rehydrated strawberries was compared with that of fresh strawberries and thawed strawberries by measuring the maximum applied load (g). As seen in Table 5, the values for the rehydrated strawberries were very far from the maximum load range for fresh strawberries. The maximum load intervals were obtained with 95% confidence and are shown in Figure 10. On the other hand, the loads for the rehydrated strawberries were within the confidence interval of values for thawed strawberries, which indicates that there was no difference between the textures of the rehydrated strawberries and the thawed strawberries.

Finally, when analyzing the color and texture of the rehydrated strawberries, there were no differences compared with the thawed strawberries; thus, freeze-drying is a good option for the long-term preservation of strawberries without the need to perform a series of cold treatments.

## 4. Conclusions

Micro-perforations, made using a CO_2_ laser, had a significant influence on the primary freeze-drying times of strawberries, reducing the treatment time by 20% when five micro-perforations were made. This time reduction may have been due to the effect on the mass transfer resistance of the dried fraction of the strawberry tissue during the freeze-drying process. This conclusion is supported by scanning electron microscopy (SEM) images that allowed us to observe, in detail, the pore size of the perforation wall, which showed that the tissues were sufficiently undamaged by calcination from the laser beam, allowing for sublimated vapor flow. The quality parameters used to evaluate the strawberries did not show significant differences when comparing frozen/thawed fruits with freeze-dried/rehydrated fruits. The experiments conducted in this study showed that freeze-drying may efficiently compete with freezing technology for the processing of whole strawberries.

We recommend further experiments that include perforating strawberries in random locations on the fruit with the aim of developing production-scale application. This should not be difficult since CO_2_ laser perforation is a well-established technology in many other fields. Future work in this direction should advance to pilot tests to obtain the information needed to scale up production methods and perform a technical–economic study.

## Figures and Tables

**Table 1:** CO_2_-laser parameter settings.

Parameter	Value
Speed	100 [cm/seg]
Power	50% of 100 [W]
Pulses	600 pulses of 1 [ms]
Frequency	50 KHz
Pattern for 5 perforations	
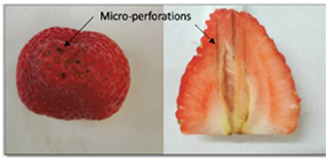	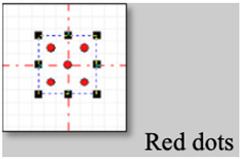

**Table 2:** Primary drying time: mean and corresponding lower and upper limits of the 95% CI. The values were obtained from the data in Figure 4, obtained from regressions, upper and lower lines at the intersection with the line of 10% Pressure Increase.

Treatment	Lower Limit	Mean	Upper Limit
Without micro-perforations	25.3	26.7 ^a^	27.1
With 5 micro-perforations	21.4	22.3 ^b^	24.1
With 8 micro-perforations	20.5	21.3 ^b^	23.6

Means with different superscripts within a column differ significantly (*p* < 0.05)

**Table 3:** Rehydration test results.

Group	Temperature °C	Weight: Fresh Fruits (g)	Weight: Freeze Dried Fruits (g)	Weight: Rehydrated Fruits (g)	Average Rehydration (%)
5 Micro-perforations	20	33.89 ± 3.1 ^a^	3.65 ± 0.51 ^a^	9.49 ± 1.9 ^a^	28% ± 8% ^a^
	2	32.97 ± 3.1 ^a^	3.72 ± 0.77 ^a^	10.78 ± 0.92 ^a^	33% ± 6% ^a^
Without micro-perforations	20	28.43 ± 2.3 ^b^	2.74 ± 0.46 ^a^	6.03 ± 0.44 ^b^	21% ± 2% ^b^
	2	33.83 ± 2.8 ^a^	3.47 ± 0.62 ^a^	7.48 ± 1.51 ^b^	22% ± 3% ^b^

Means with different superscripts within a column differ significantly (*p* < 0.05).

**Table 4:** Color test results.

Strawberry Type	Lightness (L*)	Redness (a*)	Yellowness (b*)	Hue [°]	Chroma
Fresh	34.26 ± 0.61 ^a^	32.97 ± 0.93 ^a^	18.73 ± 1.04 ^a^	29.60	37.92
Thawed	34.41 ± 1.16 ^b^	29.42 ± 0.78 ^b^	20.77 ± 1.07 ^b^	35.22	36.01
Rehydrated at 20 [°C] with 5 micro-perf.	34.59 ± 2.17 ^a,b^	28.85 ± 1.79 ^b^	22.71 ± 2.02 ^b^	38.21	36.71
Rehydred at 2 [°C] with 5 micro-perf.	38.43 ± 3.30 ^a,b^	32.17 ± 2.39 ^a,b^	25.02 ± 2.34 ^a,b^	37.87	40.76
Rehydrated at 20 [°C] without micro-perf.	35.51 ± 2.47 ^a,b^	25.87 ± 2.59 ^b^	24.26 ± 2.89 ^b^	43.16	35.46
Rehydrated at 2 [°C] without micro-perf.	33.42 ± 1.37 ^a,b^	27.64 ± 2.35 ^b^	20.17 ± 2.10 ^a,b^	36.87	34.22

Means with different superscripts within a column differ significantly (*p* < 0.05).

**Table 5:** Texture test results.

	Maximum Load Intervals [g]		
Strawberry Type	Min. Value	Medium Value	Max. Value
Fresh	817.56	1062.62	1307.68
Thawed	108.32	172.27	236.21
Rehydrated at 2 °C without micro-perf.	89.95	112.45	134.95
Rehydrated at 20 °C without micro-perf.	76.63	99.12	121.62
Rehydrated at 2 °C with 5 micro-perf.	81.43	115.15	148.87
Rehydrated at 20 °C with 5 micro-perf.	88.73	119.49	150.25

## Data Availability

The original contributions presented in the study are included in the article, further inquiries can be directed to the corresponding author.
